# Real-world experience utilizing the nuvision 4D intracardiac echocardiography catheter for left atrial appendage closure

**DOI:** 10.1038/s41598-024-60692-5

**Published:** 2024-05-24

**Authors:** Alex Adams, Riaz Mahmood, Nivedha Balaji, Priyadarshini Dixit, Shalabh Chandra, David Weisman

**Affiliations:** 1 Graduate Medical Education, Cardiovascular Disease Fellowship, Northeast Georgia Medical Center, 743 Spring Street NE, Suite 710, 30501 Gainesville, GA Georgia; 2 Graduate Medical Education, Internal Medicine Residency, Northeast Georgia Medical Center, Gainesville, GA Georgia; 3 Georgia Heart Institute, Northeast Georgia Health System, Gainesville, Georgia; 4 Jupiter Medical Center, Jupiter, FL USA

**Keywords:** Atrial fibrillation, Left atrial appendage occlusion, Intracardiac echocardiography, Stroke prevention, Cardiac device therapy, Cardiology, Interventional cardiology

## Abstract

Transesophageal echocardiography (TEE) has been the preferred imaging modality to help guide left atrial appendage closure. Newer technologies such as the Nuvision 4D Intracardiac echocardiography (ICE) catheter allow for real-time 3D imaging of cardiac anatomy. There are no direct comparison studies for procedural imaging between TEE and 4D ICE. To evaluate the performance and safety of left atrial appendage (LAA) closure procedures with the Watchman FLX and Amulet, guided by the Nuvision 4D ICE Catheter. This retrospective observational analysis was conducted on institutional LAAO National Cardiovascular Data Registry from January 2022 to March 2023. Patients had undergone LAA closure procedures with the Watchman FLX or Amulet device guided by TEE or a 4D ICE Catheter. The primary outcome evaluated was successful LAAO device placement. A total of 121 patients underwent LAAO device placement with 46 (38.0%) patients guided by 4D ICE during LAAO implantation. The 4D ICE group had a shorter procedural time compared with TEE guidance. Post procedural 45-day TEE post implant was also comparable for both groups with no patients in either group having incomplete closure of the left atrial appendage and peri-device leak > 5 mm. No device related complications (device related access, stroke, or pericardial effusion) occurred in either group at follow-up. There was no significant difference in device implant success or post procedural outcomes at 45 days in either the TEE or 4D ICE group. However, there was a noticeable improvement in procedural time with the 4D ICE catheter.

## Introduction

Atrial fibrillation (AF) is the most common arrhythmia worldwide^1^. AF is associated with an increased risk for ischemic strokes of up to 5% each year with almost 90% of the thrombi originating from the left atrial appendage^1–3^. The current gold standard against systemic thromboembolism for patients with AF is oral anticoagulation with either direct oral anticoagulants (DOACs) or vitamin K antagonists (VKAs)^2,4^.

Direct oral anticoagulants, such as dabigatran, rivaroxaban, apixaban, and edoxaban, are associated with a reduction in stroke, systemic embolism, hemorrhagic stroke, and mortality. DOACs have largely replaced the use of vitamin K antagonists, such as warfarin. VKAs are limited by the various diet-drug interactions, narrow therapeutic index, and frequent blood level monitoring^5^. Absolute contraindications to anticoagulation (intracranial mass/hemorrhage) or relative contraindications (dementia, gastrointestinal hemorrhage, anemia, thrombocytopenia) have prevented patients from having the ability to safely protect themselves from stroke in AF, but now alternative options exist to occlude the left atrial appendage subsequently decreasing embolic risk^6^.

Left atrial appendage (LAA) occlusion devices are supplanting oral anticoagulants in high risk patients due to an increased risk of hemorrhage, medication noncompliance, narrow therapeutic window associated with oral anticoagulants^7^. Both the PREVAIL and PROTECT-AF trials have demonstrated LAAO to be noninferior to VKAs in preventing all-cause stroke, systemic embolism, and cardiovascular death^5,8^. The PRAGUE-17 trial demonstrated that LAA occlusion when compared to DOACs was noninferior in preventing all-cause stroke, systemic embolism, cardiovascular death, clinically significant bleeding, and procedure-related complications^5^.

Transesophageal echocardiography (TEE) has long played an important role in cardiac procedures, both structural and cardiac electrophysiology, to help guide crucial aspects of the case, identify relevant anatomy, exclude thrombus, and evaluate for post-procedural complications. It is currently the gold standard imaging modality used for left atrial appendage evaluation and thrombus exclusion in patients undergoing radiofrequency ablation, electrical cardioversion, or LAA occlusion device deployment^2,4,8,9^. However, TEE is associated with complications such as esophageal laceration, perforation, and hemorrhage^4,8,10^. Additionally, TEE frequently requires general anesthesia and endotracheal intubation, which increases the risk of pulmonary and gastroesophageal complications, as well as patient discomfort^4,8,9^. Furthermore, TEE necessitates that a cardiac anesthesiologist or a general cardiologist be present during the procedure to manipulate the echocardiography probe for image acquisition in order to complete the procedure^11^.

The potential advantages of procedures guided by ICE compared with TEE is that these procedures allow for a single operator and obviate the requirement for general anesthesia. Most operators performing interventional cardiac procedures already have a wealth of experience utilizing this technology. Two-dimensional (2D) ICE catheters were used to assess thrombus mobility, delineate thrombotic structural changes, measure left atrial appendage measurements, and provide intraprocedural guidance^3,9^. One of the first studies evaluating ICE guided intracardiac LAA occlusion, utilized a 2D 10Fr AcuNav ICE catheter, device deployment was successful but was noted to have a 6.6% major safety event rate^12^. The likely limitations of initial 2D ICE were the fixed 45-degree field of view that lacked the ability for multiplanar imaging^12^. A meta-analysis of 8 observation studies comparing ICE and TEE (TEE = 1733 vs. ICE = 578 total patients) compared procedural time, complications, and procedural success and were found to be similar between both groups^13^.

Real-time three-dimensional (3D) ICE also known as four-dimensional (4D) ICE provides improved characterization of the left atrial appendage anatomy in multiple views, accurate volumetric measurements and spatial orientation in real time, and circumferential peri-valvular and peri-device flow when used with color doppler to assess for post-LAAO device deployment leakage and effusion^9^. ICE is associated with lower implant periprocedural complication rates, and has been shown to reduce preparation time for LAA occlusion device placement, shorten overall hospital length of stay, and reduce the rate of post-procedure fever when compared to TEE^4,8,9^. ICE catheters are directed into the right atrium from a femoral venous access point allowing for conscious sedation and local anesthesia without tracheal intubation^14^. Unlike TEE guided procedures, ICE can be performed by a single operator without the need for an additional imager. Additionally, ICE-guided LAA occlusion procedure does not increase the risk of iatrogenic atrial septal defects^10^. ICE-guided LAA occlusion can be used in patients with esophageal tumors or varices who are contraindicated to undergo TEE and elderly patients who cannot tolerate general anesthesia^8^.

There is limited data on the efficacy of this new 4D ICE Catheter, one such observation study evaluated catheter used in 7 cases used for pulmonary vein isolation for an atrial fibrillation ablation and 2 cases for LAA occluder devices without complications^15^, but data is limited on how this novel technology compares to the gold standard TEE. There is currently no literature that evaluates this novel technology in a randomized clinical trial to TEE. The goal of this study was to evaluate the performance and safety of Nuvision 4D ICE Catheter for guided LAAO procedures using Watchman FLX and Amulet devices.

## Methods

This was a retrospective observational single center analysis of patients undergoing percutaneous left atrial appendage closure. We identified patients that underwent LAA occlusion procedures from our institutional National Cardiovascular LAAO Data Registry from January 2022 to March 2023. To reduce inter-operator variability, the analysis was limited to procedures performed by three experienced implanting electrophysiologists with the Watchman FLX or Amulet device guided by either TEE or the Nuvision 4D ICE Catheter.

Pre-imaging modalities for evaluation of LAA prior to procedure included TEE, Coronary CT Angiography (CCTA) left atrial appendage protocol were used to evaluate the LAA for sizing of the device with the largest diameter obtained from the 0, 45, 90 and 135 degrees on TEE or the perimeter of landing zone measurement from CCTA for device implant. Intraprocedural TEE or 4D ICE further measured the LAA size to ensure accuracy and confirm measurements prior to implant.

**Figure 1:**
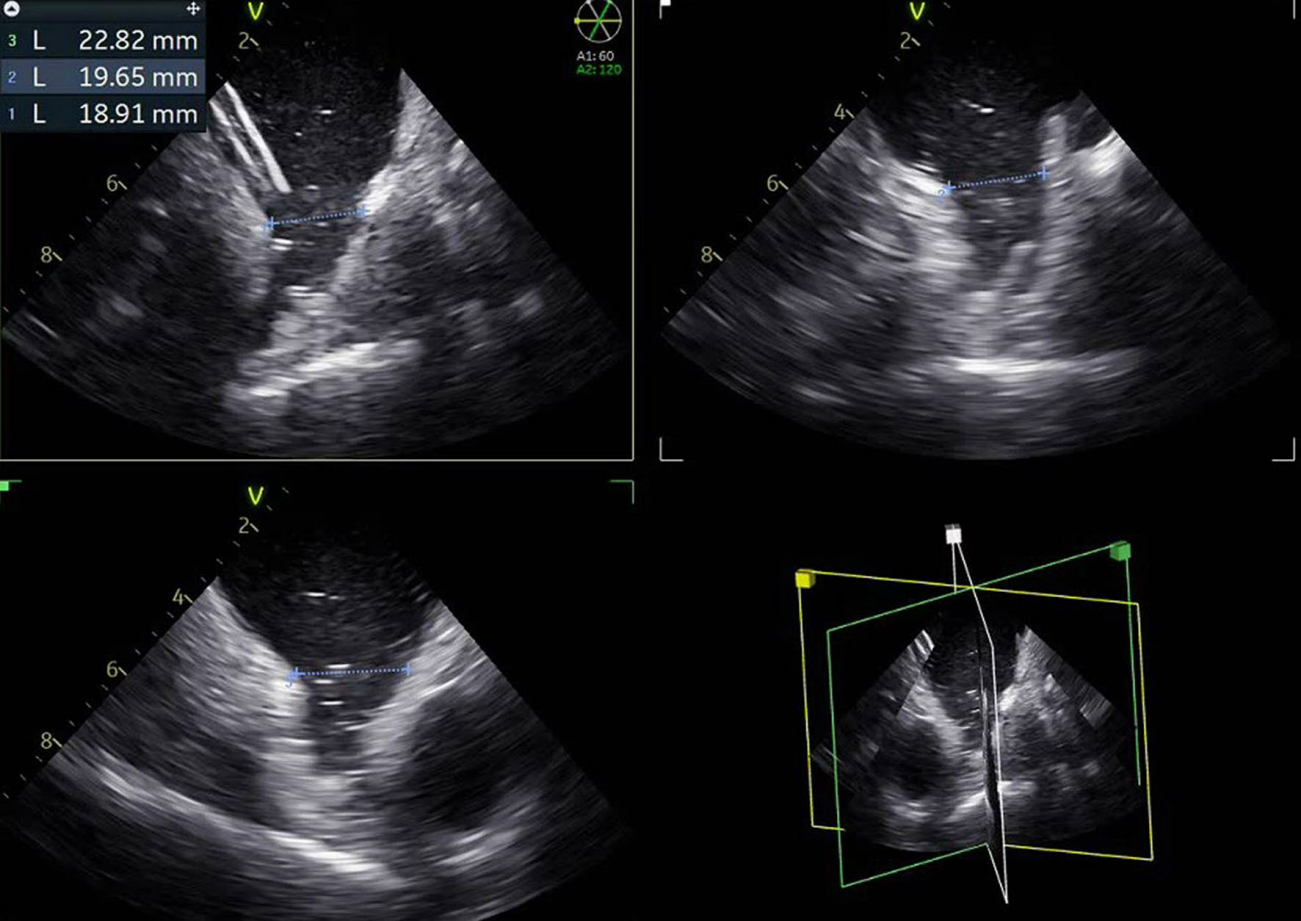
4D ICE Catheter assessment of LAA sizing for device selection.

**Figure 2:**
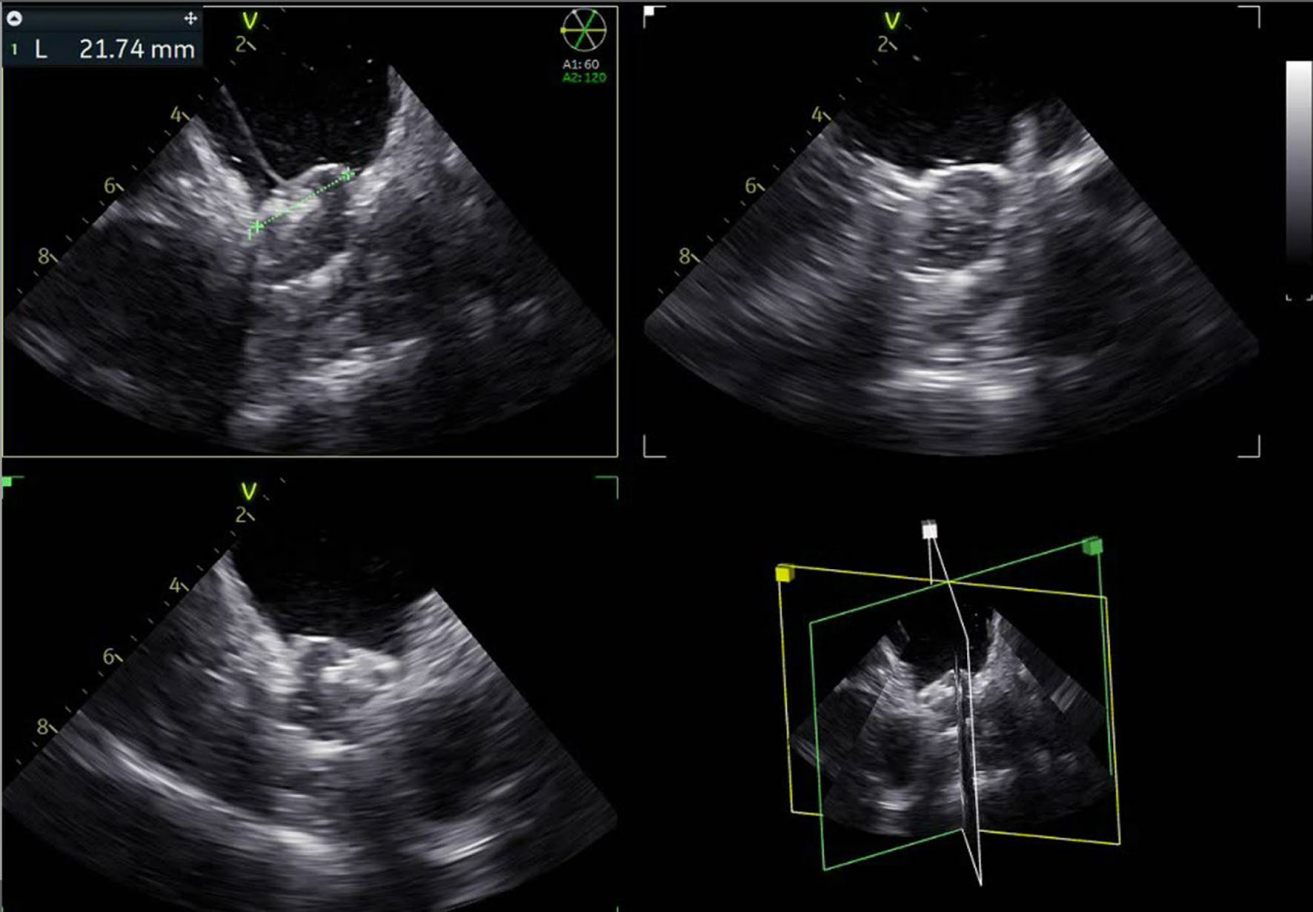
4D-ICE Catheter guidance of LAAO implant.

**Figure 3:**
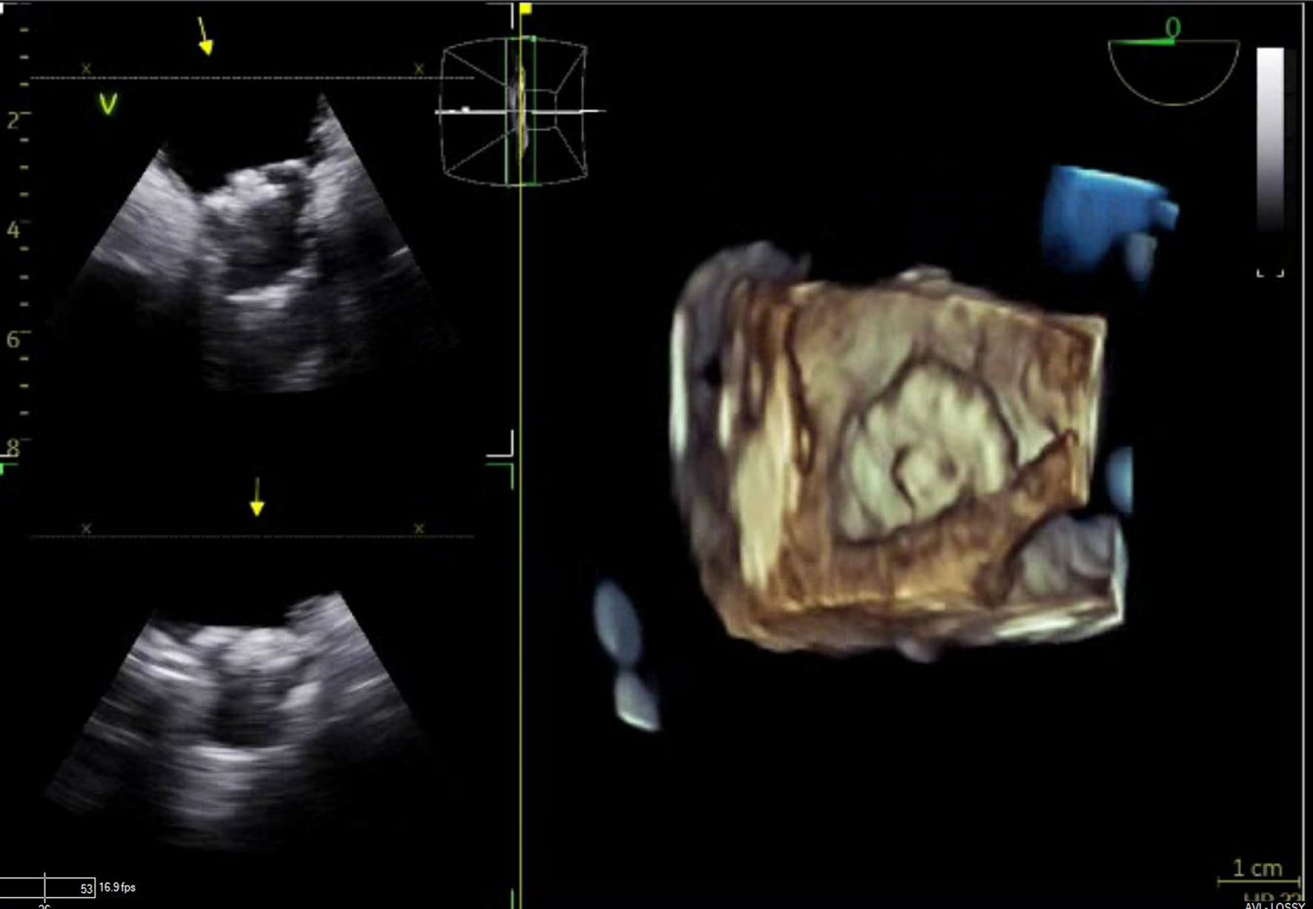
4D ICE Catheter post implant evaluation.

4D ICE Catheters were used to exclude LAA thrombus, guide transseptal puncture, size the left atrial appendage for appropriate device selection (Fig. 1), guide cannulation of LAA (Fig. 2), and device deployment. Post implant measurements were successfully taken to ensure well seated devices (Fig. 3) without peri-device leaks, and to confirm the absence of pericardial effusion at the end of the procedure. Follow up data was collected at > 45 days and TEE imaging was also performed. One patient was unable to have TEE performed at > 45 day follow up and alternatively Cardiac Computed Tomography angiography was performed to evaluate for successful LAA occlusion.

Inclusion criteria included adults over the age of 18, procedure was performed by one of the three electrophysiologists, and successful implant of a left atrial occluder device. Patients were excluded during this period whose left atrial appendage anatomy was unsuitable for device deployment due to shallow depth, LAA morphology, or the finding of intraprocedural LAA thrombus.

The primary outcome evaluated was successful LAAO device placement. A successful LAA implant was defined as complete LAA occlusion, defined by Manufacturer specific implant criteria (PASS for Watchman FLX/ CLOSE for Amulet, Table 1). TEE imaging without evidence of peri-device leak, which was defined as peri-device leak > 5 mm. The secondary safety endpoint was defined as no device related complications in terms of device related access, stroke, mortality, or post-procedure pericardial effusion.

**Table 1:** Criteria for PASS and CLOSE.

Device specific implant criteria	
Watchman FLX	Amulet
**P**—**Plane:** of maximum diameter of the Closure Device should be at, or just distal to, the LAA ostium, where possible, while meeting all other PASS criteria	**C—**At least 2/3 of the device lobe should be distal to the left **Circumflex** artery on echocardiography
**A**—**Anchor**: Gently pull back then release deployment knob to visualize movement of Closure Device and LAA together	**L**—The Device **Lobe** should be slightly compressed and have good apposition to the left atrial appendage wall
**S**—**Size** (compression): Measure plane of maximum diameter of Closure Device	**O**—The disc must be **Separated** from the lobe
**S**—**Seal**: Ensure all lobes of the LAA are distal to Closure Device and sealed (e.g., no leak > 5 mm)	**S**—The **Orientation** of the device lobe must be in line with the axis of the intended landing zone in the left atrial appendage
	**E**—The disc will have a concave (**Elliptica**l) shape

Significant values are in [bold].

All methods were carried out in accordance with relevant guidelines and regulations. All experimental protocols were approved by Breanau University IRB. The same institution waived need for informed consent in the manuscript.

### Statistical analysis

Statistical analyses were performed using JMP Pro software version 17 (Cary, NC: SAS Institute Inc.). For categorical variables, displayed as values (percentages), were compared using Fisher's exact test. For continuous variables, groups were displayed as means with standard deviation and compared with t-tests. All tests were two-sided. A p value of less than 0.05 was considered statistically significant.

The datasets generated and/or analyzed during the current study are available in attached supplementary file.

## Results

**Figure 4:**
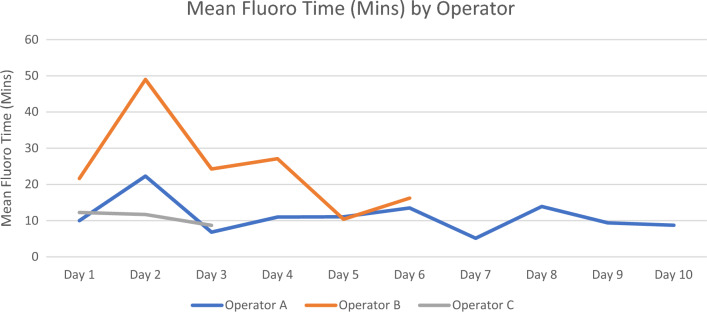
Shows the mean fluoroscopy times by operator over time.

A total of 121 patients were reviewed with 46 (38.0%) patients undergoing 4D ICE catheter guided LAA occluder implantation. The overall average age was 76.2 ± 7.0, 74 (61.1%) were males, mean body mass index (BMI) was 29.7 ± 5.5, and the mean CHA_2_DS_2_-VASc was 4. There were no statistically significant differences for age, gender, BMI, CHA_2_DS_2_-VASc score, history of Coronary Artery Disease, Congestive Heart Failure, and Peripheral Artery Disease, Table 2. 4D ICE group had a shorter procedural time compared with TEE guidance, but increased fluoroscopy times, Table 2. Mean daily fluoroscopy times declined following Day 2, Fig. 4. Post procedural 45-day TEE follow was also comparable for both groups with no patient’s in either group having incomplete closure of the left atrial appendage and peri-device leak > 5 mm. No device related complications occurred in either group in terms of device related access, stroke, mortality, or pericardial effusion.

**Table 2:** Demographics and Procedural Time for 4D ICE and TEE.

	4D ICE (N = 46)	TEE (N = 75)	*p* value
Age	76.4 ± 6.7	76.1 ± 7.2	0.78
Gender (Male)	30 (65.2%)	44 (58.7%)	0.47
BMI	30.8 ± 4.9	29.0 ± 5.7	0.07
CHA_2_DS_2_-VASc	4.1 ± 1.2	4.3 ± 1.4	0.54
HAS-BLED	**4.3 ± 1.0**	**3.0 ± 0.81**	**< 0.001**
Coronary artery disease	23 (50.0%)	29 (38.7%)	0.22
Congestive heart failure	19 (41.3%)	24 (32.0%)	0.30
Peripheral artery disease	6 (13.0%)	4 (5.3%)	0.14
Contrast (mL)	22.1 ± 8.7	22.5 ± 11.0	0.83
Fluoroscopy time (Minutes)	**14.1 ± 9.4**	**9.1 ± 6.9**	**0.002**
Device type- watchman FLX	**78.26%**	**100%**	**< 0.001**
Device size (Percentage)	**31 (26.09%)**	**27 (33.33%)**	**< 0.001**
Procedural time (Minutes)	**66.7 ± 34.4**	**90.2 ± 24.3**	**< 0.001**
Peri-device Leak > 45 days	**0 (0.0%)**	**0 (0.0%)**	**< 0.001**

Significant values are in [bold].

## Discussion

The emergence of the 4D ICE Catheter has introduced a novel way to guide LAA occlusion procedures with the capability of 2D/4D color doppler flow, real time multiplanar visualization with 90° × 90° field of view, allowing for a front on view of the LAA with ability to measure long and short axis simultaneously. Our retrospective observational study demonstrates that 4D ICE Catheter imaging is effective and safe when compared with TEE for LAAO device placement. Both groups have similar baseline characteristics and relevant comorbidities, increasing the probability that the results are generalizable and reproducible.

**Figure 5:**
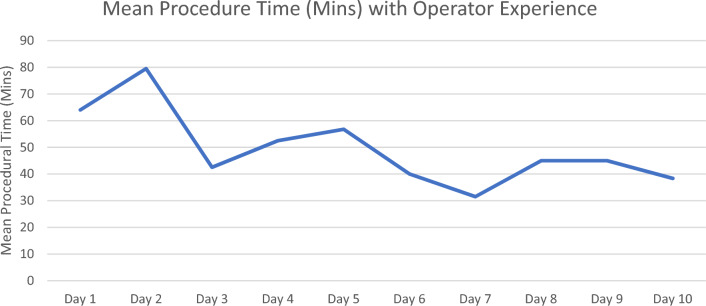
Shows the downward trend for procedural time with operator experience.

There was a significant decrease in mean procedural time, 23.5 min. Previous studies comparing ICE and TEE have demonstrated comparable procedure times (access to closure)^16^, however; this new technology safely and effectively shortens procedure times. Procedure times are likely shorter because of the ability to view the LAA in multiple orthogonal views simultaneously. Additionally, only a single operator is needed to maneuver the 4D ICE catheter, allowing for quicker image acquisition and catheter manipulation. Although procedure times were shorter, this likely underestimates the amount of time from access to closure with the 4D ICE catheter as there was a learning curve with this new device and overall trend to shorter procedure times with subsequent use. The average procedure time for one experienced operator was 41 min after a 12-case learning curve to adjust to the new technology. Figure 5 shows the decreasing trend in procedural time for this operator. We would expect to see a greater than 24 min decrease in procedural time after one year of experience. Future studies should further evaluate procedural time after allowing for a fixed learning curve exclusion for the new device.

Fluoroscopy time was noted to be longer with the use of 4D ICE catheters. However, following Procedural Day 2, as each procedural day had multiple stacked LAAO occlusion cases per operator, fluoroscopy times declined over time. The nature for increased fluoroscopy time is likely attributed to the learning curve associated with this new technology and there was a trend of shorter procedural time per operator when adjusting for a learning curve for the new technology. One previous study of ICE vs TEE evaluated contrast media use and noted that when comparing groups, significantly larger amounts of contrast media usage were noted in ICE catheter cases (ICE 40 ml ± 23 mL vs TEE 30 mL ± 6 ml)^17^. In our experience we did not find that there was a significant difference between contrast media usage in either group (4D ICE 22.1 ± 8.7 mL vs. TEE 22.5 ± 11.0 mL), with slightly decreased contrast media usage in the 4D ICE group.

Future comparative cost analysis would help to validate the economics of 4D ICE utilization. One previous study comparing ICE vs TEE financial costs, demonstrated that hospital charges were increased in the ICE group due to catheter cost, but professional fees were significantly higher in the TEE group^18^. When comparing the overall or combined costs of both technologies, professional fees and hospital charges were similar in the ICE and TEE groups ($79,020 ± 8241 vs. 77,147 ± 10,941; *p* = 0.15)^18^. When taking into consideration that 4D-ICE catheters significantly shorten procedure times, even if the cost of TEE versus 4D ICE was comparable, which was not evaluated during this study, one would expect increased lab efficiency and the potential for higher daily case volumes. In our center, typical procedures days are stacked with multiple LAAO cases to improve lab efficiency. Additionally, there is no longer a need to coordinate with other service lines to provide intraprocedural imaging. Furthermore, the implementation of 4D ICE allows these imaging physicians to be utilized in other capacities.

The use of ICE catheters requires an additional venous access site which could potentially increase the risk of vascular complications (groin hematoma, AV Fistula, retroperitoneal bleeding), no increase in vascular complications was noted in our study. There were no device related access complications, stroke, mortality, or post-procedure pericardial effusion in either group.

The use of 4D ICE catheters expands the pool of patients who could benefit from LAA occlusion who were deemed poor surgical candidates due to the increased risk for general anesthesia, higher aspiration risk, and those who have esophageal pathologies (I.e. varices or strictures) who otherwise would be contraindicated for TEE. Based on the evidence from our study, 4D ICE in combination with fluoroscopy can successfully allow for LAA occluder device selection, implant, and exclusion of peri-device leak. Over the next 2–3 years, there will likely be more LAA occlusion device implants with 4D ICE compared to TEE.

### Limitations

Given the retrospective single center observation design, there is a lack of randomization. Future pragmatic studies are needed with larger sample size to adjust for any confounding variables. No safety issues were noted in either group, which could be due to a small sample size. There is always variability in procedure success and safety based on operators. We attempted to limit inter-operator variability by utilizing the same three operators across both arms. Since all three operators were experienced at LAAO device implantation, it does raise the concern if inexperienced operators would be able to have the same low complication rates and high implant success rate. Amulet occlusion device was only implanted in 10 (8%) of the total devices for the three operators. All these devices were done under guidance of 4D-ICE catheter and therefore cannot be compared directly to TEE guidance; however, we did not see any adverse events or implant failures in this group as well.

## Conclusion

The Nuvision 4D ICE Catheter provides a reasonable alternative to guide LAA Occlusion procedures without compromising success or safety. There was no significant difference in device implant success or post procedure outcomes at greater than 45 days; however, there was a noticeable improvement in procedural time with the Nuvision 4D ICE catheter.

### Supplementary Information

Supplementary Information.

## Data Availability

No datasets were generated or analysed during the current study.

## Abbreviations

LAA: Left atrial appendage
ICE: Intracardiac echocardiography (ICE)
4D: Four-dimensional
3D: Three-dimensional
2D: Two-dimensional
TEE: Transesophageal echocardiography
AF: Atrial fibrillation
VKA’s: Vitamin K antagonists
DOAC: Direct oral anticoagulation
BMI: Body mass index
