# Author Correction: Real-world experience utilizing the nuvision 4D intracardiac echocardiography catheter for left atrial appendage closure

**DOI:** 10.1038/s41598-024-68535-z

**Published:** 2024-08-01

**Authors:** Alex Adams, Riaz Mahmood, Nivedha Balaji, Priyadarshini Dixit, Shalabh Chandra, David Weisman

**Affiliations:** 1 Graduate Medical Education, Cardiovascular Disease Fellowship, Northeast Georgia Medical Center, , 743 Spring Street NE, Suite 710, 30501 Gainesville, GA Georgia; 2 Graduate Medical Education, Internal Medicine Residency, Northeast Georgia Medical Center, Gainesville, GA Georgia; 3 Georgia Heart Institute, Northeast Georgia Health System, Gainesville, Georgia; 4 Jupiter Medical Center, Jupiter, FL USA

Correction to: *Scientific Reports* 10.1038/s41598-024-60692-5, published online 24 May 2024

The original version of this Article contained an error in the order of the author names, which was incorrectly given as Alex Adams, Riaz Mahmood, Nivedha Balaji, Priyadarshini Dixit, David Weisman & Shalabh Chandra.

The original Article has been corrected.

